# Biomechanical stability of a supra-acetabular pedicle screw Internal Fixation device (INFIX) vs External Fixation and plates for vertically unstable pelvic fractures

**DOI:** 10.1186/1749-799X-7-31

**Published:** 2012-09-27

**Authors:** Jonathan M Vigdorchik, Amanda O Esquivel, Xin Jin, King H Yang, Ndidi A Onwudiwe, Rahul Vaidya

**Affiliations:** 1 Detroit Receiving Hospital, 4201 St Antoine St, Detroit, MI 48201, USA; 2 Providence Hospital and Medical Centers, 16001 West 9 Mile Road, Southfield, MI, USA; 3 Wayne State University, 540 E Canfield St, Detroit, MI, USA

## Abstract

**Background:**

We have recently developed a subcutaneous anterior pelvic fixation technique (INFIX). This internal fixator permits patients to sit, roll over in bed and lie on their sides without the cumbersome external appliances or their complications. The purpose of this study was to evaluate the biomechanical stability of this novel supraacetabular pedicle screw internal fixation construct (INFIX) and compare it to standard internal fixation and external fixation techniques in a single stance pelvic fracture model.

**Methods:**

Nine synthetic pelves with a simulated anterior posterior compression type III injury were placed into three groups (External Fixator, INFIX and Internal Fixation). Displacement, total axial stiffness, and the stiffness at the pubic symphysis and SI joint were calculated. Displacement and stiffness were compared by ANOVA with a Bonferroni adjustment for multiple comparisons

**Results:**

The mean displacement at the pubic symphysis was 20, 9 and 0.8 mm for external fixation, INFIX and internal fixation, respectively. Plate fixation was significantly stiffer than the INFIX and external Fixator (P = 0.01) at the symphysis pubis. The INFIX device was significantly stiffer than external fixation (P = 0.017) at the symphysis pubis. There was no significant difference in SI joint displacement between any of the groups.

**Conclusions:**

Anterior plate fixation is stiffer than both the INFIX and external fixation in single stance pelvic fracture model. The INFIX was stiffer than external fixation for both overall axial stiffness, and stiffness at the pubic symphysis. Combined with the presumed benefit of minimizing the complications associated with external fixation, the INFIX may be a more preferable option for temporary anterior pelvic fixation in situations where external fixation may have otherwise been used.

## Background

**Figure 1:**
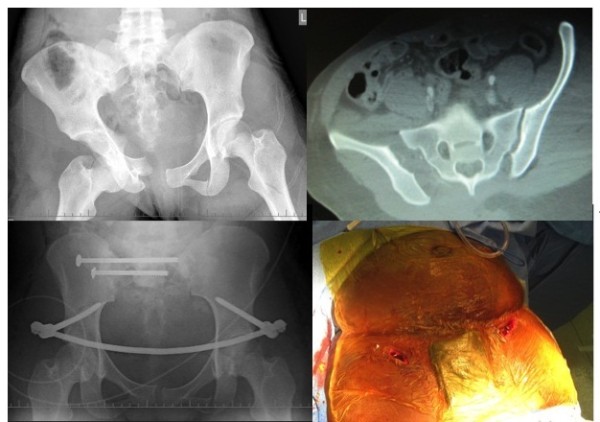
Case Example a) Xray APC 3 Pelvic injury b) CT scan showing opening of Right SI joint c) Fixation with posterior SI screws and INFIX anteriorly d) Incisions for insertion of INFIX before closure.

External fixation for unstable pelvic fractures is effective in resuscitation and reduces pelvic volume, minimizes motion between disrupted fracture surfaces or joints, allows tamponade of ongoing venous bleeding and is a fairly quick procedure [1-5]. Side to side rolling of patients, sitting and laying prone is limited with the use of pelvic external fixators. In addition the pins are always at risk for pin tract infection and require constant nursing surveillance particularly in obese patients [6]. We have recently developed a technique using the already established principles of anterior external fixation but placed subcutaneously. This technique basically involves two pedicle screws, one in each ilium placed in the supra-acetabular area connected to each other via a subcutaneous 6 mm rod tunnelled just below the belly crease, in an area called the bikini area [7]. This internal fixator (INFIX) permits patients to sit, roll over in bed and lie on their sides without the cumbersome external appliances or their complications [8] (Figure 1). The purpose of this study was to evaluate the biomechanical stability of this novel supra-acetabular pedicle screw internal fixation construct (INFIX) and compare it to standard internal fixation and external fixation techniques in a single stance pelvic fracture model. 

## Methods

**Figure 2:**
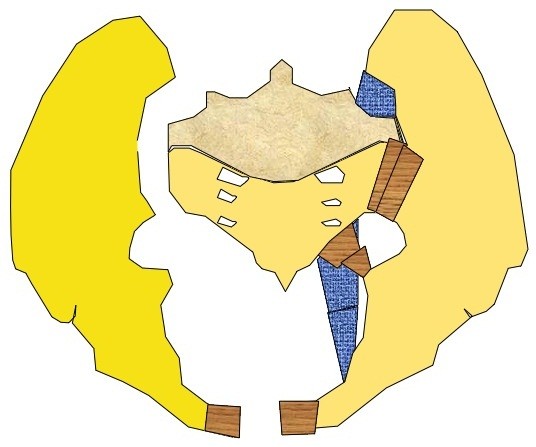
The APC III pelvis model with complete disruption of the right SI joint and Symphysis pubis.

Nine synthetic pelves (#1301-21, Sawbones, Vashon, WA) with a simulated anterior posterior compression (APC) type III (Figure 2a) injury were placed into the following groups:

1. INFIX (n = 3), by 7.0 mm × 80 mm titanium polyaxial pedicle screws and 6.0 mm stiff titanium rod (Click’X Pedicle Screw System, Synthes, West Chester, PA) (Figure 2b,c)

2. Internal fixation (n = 3), by 3.5 mm 4-hole pubic symphysis plate (Synthes, West Chester, PA)

3. External fixation (n = 3), by 2 supra-acetabular 5.0 mm Schanz pins, with an 11 mm carbon fiber rod (Synthes, West Chester, PA)

**Figure 3:**
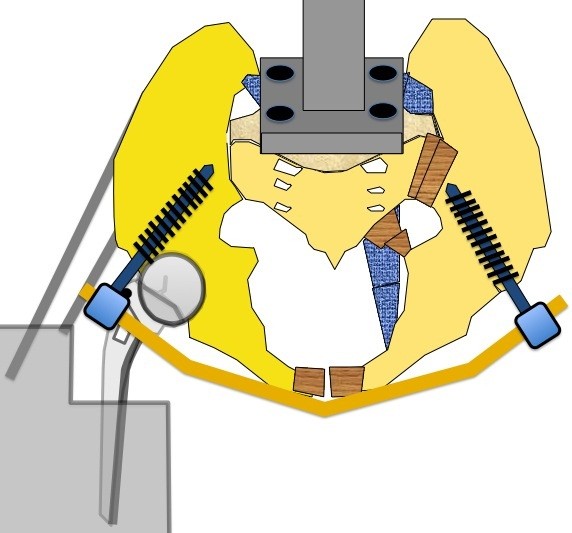
**The pelvis mounted on the apparatus with the INFIX Apparatus.** The sacrum is attached to the Instron Device and the separated hemi pelvis is fixed with wire cables to simulate the abductor muscles with the Hip prosthesis potted to simulate the single stance gait.

A single orthopaedic surgeon prepared all the specimens. A custom device was attached to the sacrum and connected to the load cell of a uni-axial servohydraulic testing device (Instron Corp, Canton, MA) through a ball and socket joint which was allowed to articulate freely (Figure 3). A unipolar hemiarthroplasty prosthesis was potted at 15 degrees adduction, to simulate a single leg stance. Each pelvis was tilted 45 degrees anteriorly and the femoral head was in contact with the acetabulum. A cable and pulley system was used to simulate abductor muscle pull on the pelvis. The abductor cables were loaded prior to each test cycle at 10 N. A compressive load was applied through the sacrum up to 200 N or 15 mm of displacement. Markers were placed at the pubic symphysis and sacroiliac (SI) joint and high-speed video was recorded at 125 frames/sec to measure displacement. Displacement, total axial stiffness, and the stiffness at the pubic symphysis and SI joint were calculated [9,10]. Displacement and stiffness were compared by ANOVA with a Bonferroni adjustment for multiple comparisons. 

## Results

**Figure 4:**
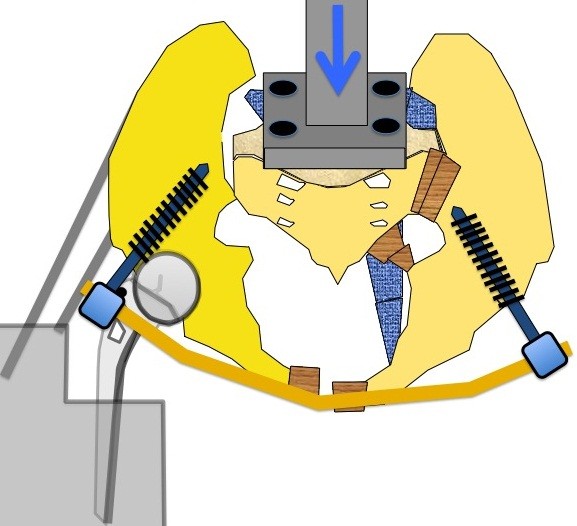
Loading of the construct with the INSTRON 200N or 15 mm.

**Figure 5:**
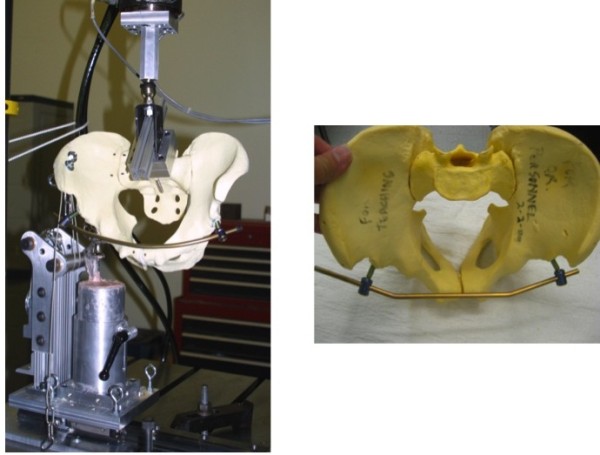
Picture of the testing apparatus.

**Figure 6:**
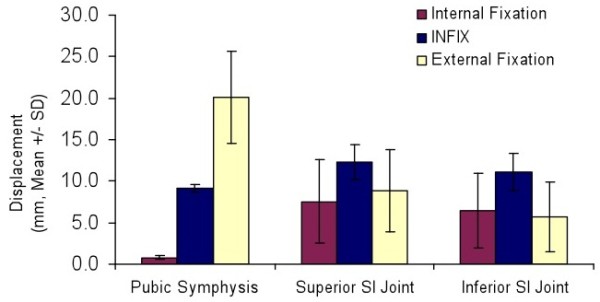
Mean displacement of each construct under axial loading.

**Figure 7:**
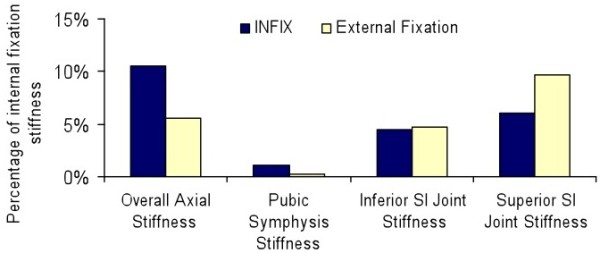
Comparison of mean stiffness of the INFIX and external fixation to internal fixation.

The mean displacement at the pubic symphysis was 20, 9 and 0.8 mm for external fixation, INFIX and internal fixation, respectively (Figure 4). This difference was statistically significant for the INFIX device when compared to external fixation (*P* = 0.017). There was also a significant difference between internal fixation and external fixation (*P* = 0.01). There was no significant difference in SI joint displacement between any of the groups. In terms of stiffness (N/m), internal fixation was significantly (*P* < 0.05) stiffer than either construct at the pubic symphysis. There was no significant difference between the stiffness of the INFIX and the external fixation device. Stiffness of the INFIX and external fixation constructs were then compared as a percentage of internal fixation stiffness (Figure 5). Although, not significantly different, the INFIX system was almost twice as stiff as external fixation (10.5% vs 5.6%) in overall stiffness and also stiffer at the pubic symphysis (1.1% vs 0.3%) the area of most clinical importance for this type of fixation. At the superior portion of the anterior SI joint, external fixation was stiffer than the INFIX (9.7% vs 6%) (Figures 6 and 7).

## Discussion

The purpose of this investigation was to compare the relative stiffness of three anterior pelvic fixation constructs. A significant difference was observed between the constructs for pubic symphysis displacement. We found that the symphyseal plate (3.5 mm 4-hole pubic symphysis plate Synthes, West Chester, PA) was significantly stiffer than the INFIX or the external fixator (2 supra-acetabular 5.0 mm Schanz pins, with an 11 mm carbon fiber rod Synthes, West Chester, PA) with this single leg stance pelvic model at the symphysis pubis (p < 0.05). The INFIX construct allowed less than half the displacement at the symphysis pubis (9 vs 20 mm) when compared to the external fixator (p < 017). When we compared stiffness there was a trend for the INFIX to be stiffer at the symphysis pubis verses the external fixator but this was not significant. The external fixator had slightly less displacement at the SI joint verses the INFIX (not significant). There was no significant difference in stiffness between any of the threee constructs at the SI joint. This is consistent with previous studies that have indicated that there is wide variability between fixation devices at this point [10,11]. Many previous studies have been performed testing internal fixation techniques for pelvic ring injuries with single or double leg stance. We chose a single-leg stance model, as this was the most unstable scenario, with greater shear, bending and rotational forces than a double-leg stance and has been postulated to be more relevant to the clinical application [12,13]. Other studies have used strain gauges, inclinometers, linear voltage transducers, or electromagnetic motion sensors to measure displacements, rotations, and 3-dimensional motions [9-12,14-19]. We chose high-speed video because it allowed us to evaluate in real-time the complex motions across the pelvis, and gave us an accurate (up to 0.8mm) detection of displacement to measure the stiffness of each construct. It is difficult to extrapolate data across studies as many different fixation methods and loading techniques were examined: transiliac plates, 1, 2, or 3 sacroiliac screws, transiliac rods, and tension band plates have all been examined in single [12,13,20-22] and double leg stance [9,14,16-18,23] models with varying loads, from 250-2000N. Different injury patterns were also tested, with or without stabilization of the anterior pelvis [13,14,16,17]. Between studies and between constructs, no significant differences in stability were shown. However, all methods of fixation are inferior to the intact pelvis [9,16]. A limitation to this study was the small sample size for each construct; however, there was a significant difference observed in pubic symphysis gapping. We used a synthetic pelvic model which has been used in several pelvic biomechanic studies because there is uniform material for fixation and testing [2,11]. Although cadaver pelvises are optimal it would be difficult to compare the two pin external fixator and the INFIX construct in the same model due to the same location of screw insertion and in different models because of the variability between specimens. Unstable type C or APC III injuries are recommended to have both anterior and posterior fixation (24). However, this biomechanical test model represents the worst-case scenario in terms of force transmission that would be seen clinically and provides a repeatable, previously used biomechanical testing method to compare the stiffness of the constructs.

## Conclusion

The data show that both the INFIX and the external fixator are significantly weaker than internal fixation at the pubic symphysis. The INFIX was stiffer than external fixation for both overall axial stiffness, and stiffness at the pubic symphysis, the area of most clinical importance for this type of fixation. Combined with the presumed benefit of minimizing the complications associated with external fixation, the INFIX may be a more preferable option for temporary anterior pelvic fixation in situations where external fixation may have otherwise been used. Future improvements to the design may help to increase the stability of the construct.

## Abbreviations

INFIX: Internal fixator; APC: Anterior posterior compression; ANOVA: Analysis of variance; SI Joint: Sacroiliac joint.

## Misc

Jonathan M Vigdorchik, Amanda O Esquivel, Xin Jin, King H Yang, Ndidi A Onwudiwe and Rahul Vaidya contributed equally to this work.

## Competing interests

Dr. R Vaidya Corresponding author is developing an FDA approved product with Stryker Corporation which has not been manufactured yet or produced. That is not the device in this study as these are just common pedicle screws and rods that are in any spine set. No other author has any competing interests.

## Authors’ contributions

Role of each author - JV – First author, Participated in developing the study design, implanted the specimens, worked on writing the paper, and performed testing - AE - Second author participated in developing the study design, worked on writing the paper, testing and result evaluation - XJ: Helped with study design, testing, result evaluation and statistics - KY: Helped with study design, testing, result evaluation and statistics - NO: Participated in writing the manuscript, formatting, statistics checks and submitting of the manuscript RV: Corresponding Author, Participated in. All authors read and approved the final manuscript.

## Author details

^1^Detroit Receiving Hospital, 4201 St Antoine St, Detroit, MI 48201, USA.^2^Providence Hospital and Medical Centers, 16001 West 9 Mile Road, Southfield, MI, USA. ^3^Wayne State University, 540 E Canfield St, Detroit, MI, USA.
